# Supplementary material for: Metagenomics-based analysis of mobile genetic elements and antibiotic/metal resistance genes carried by treated wastewater
Source: PeerJ. 2025 Jul 23;13:e19682. doi: 10.7717/peerj.19682 (PMC12296574; doi:10.7717/peerj.19682)
Supplement: Supplemental Information 1 [file peerj-13-19682-s001.docx]

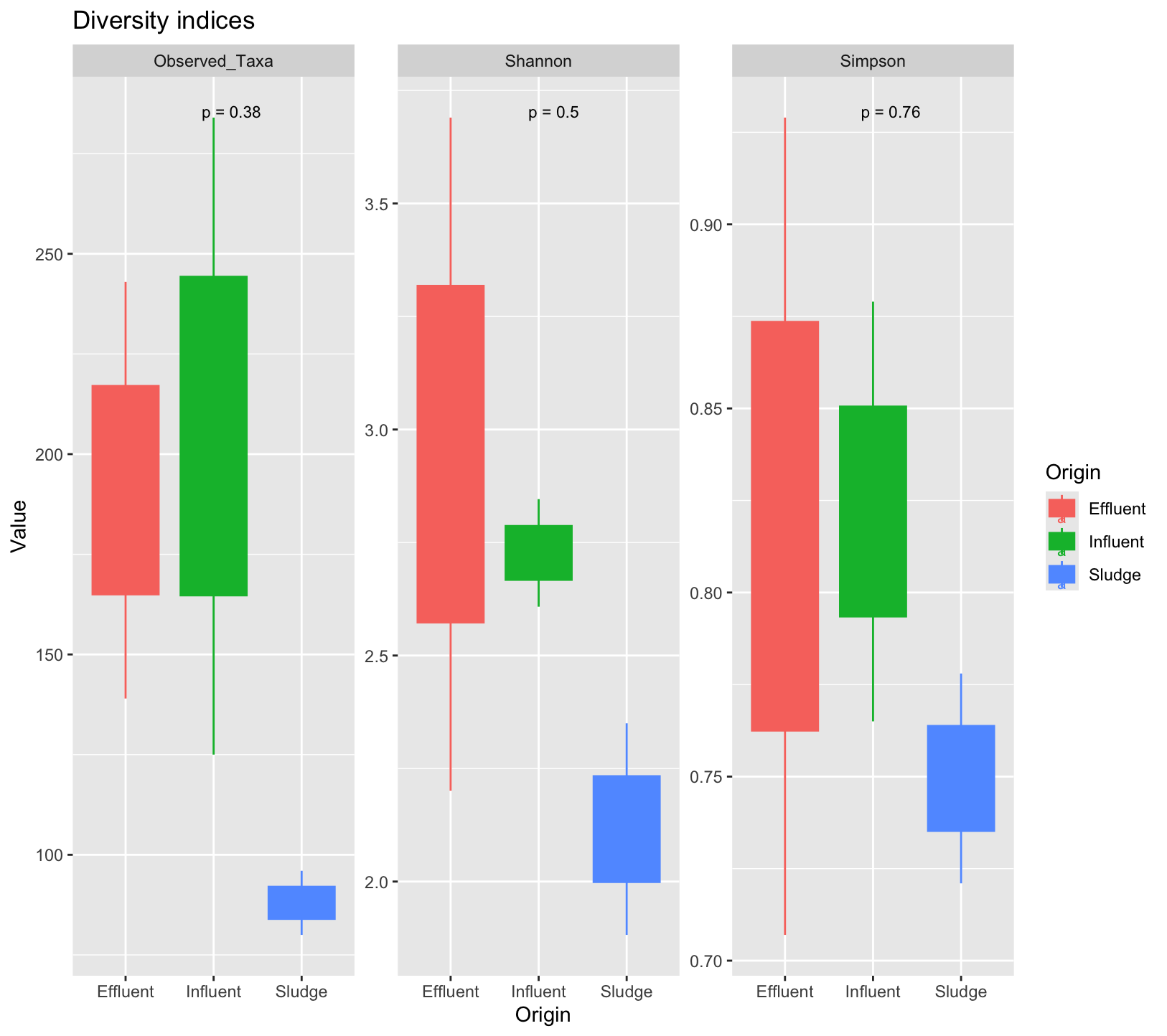


**Figure S1:** Richness and diversity analyses in the three different Charguia WWTP sites : Influent, Sludge and Effluent.


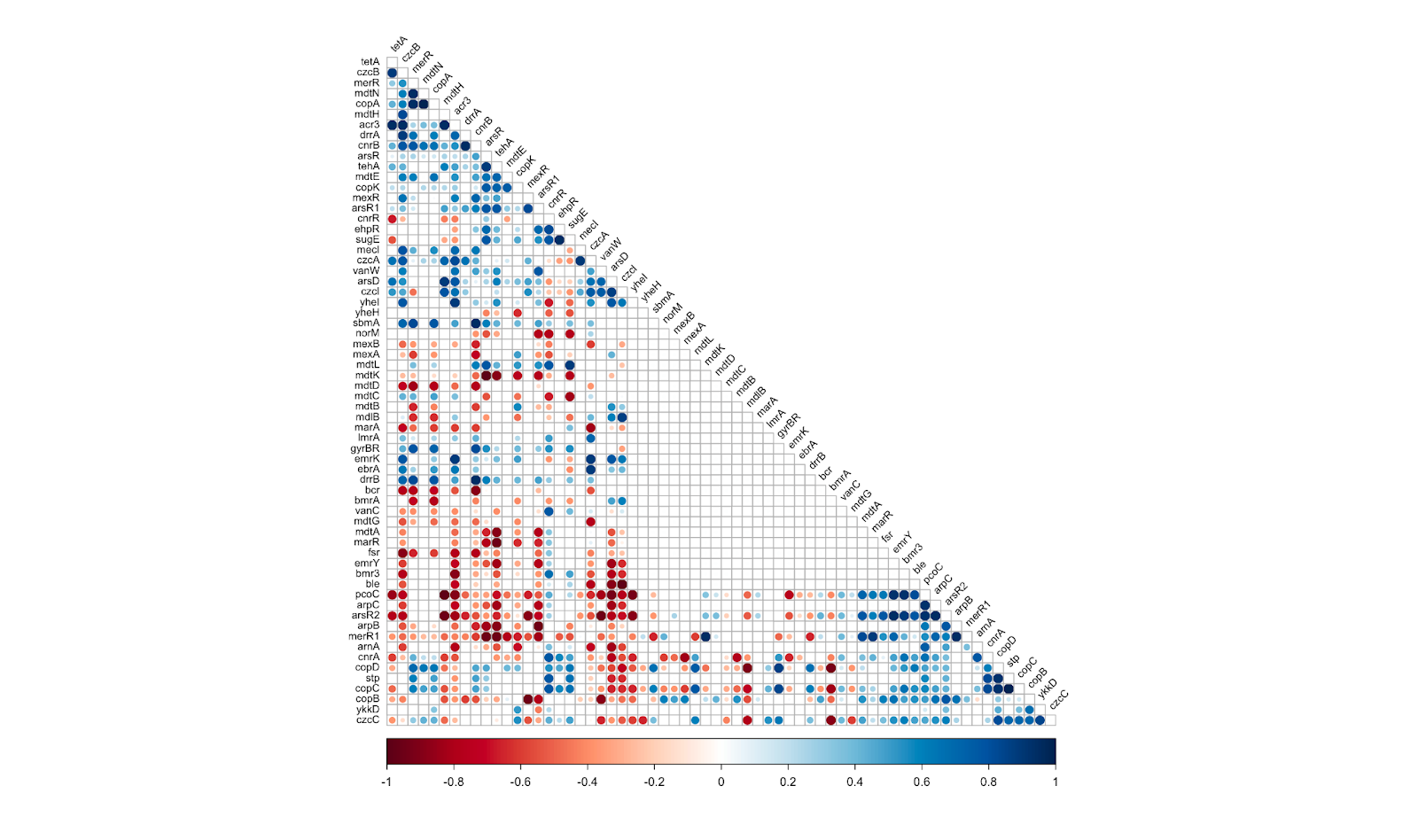


**Figure S2:** Correlation plot of the ARGs and MRGs association of Charguia WWTP samples.

*P-value* = 0.05; r<0: negative correlation; r>0: positive correlation; blank box: no significant correlation or cross correlation (ARG-ARG ; MRG-MRG).

**Table S1:** Summary table of metagenomic data of collected samples from different sites within

WWTP (Wastewater Treatment Plant) of Charguia

| **Samples** | **Raw** | **Trimmed/Filtered** | **Number of contigs** |
| --- | --- | --- | --- |
| INF1 | 15.9 | 13.5 | 443035 |
| SLD1 | 17.3 | 15.5 | 249988 |
| EFF1 | 18.5 | 16 | 560719 |
| INF2 | 9.7 | 8.4 | 400179 |
| SLD2 | 21.6 | 18.6 | 595250 |
| EFF2 | 15.6 | 13.3 | 386048 |

(Raw and Trimmed/Filtered reads: Million sequences)
