# Supplementary figures and images for: Metagenomics-based analysis of mobile genetic elements and antibiotic/metal resistance genes carried by treated wastewater
Source: PeerJ. 2025 Jul 23;13:e19682. doi: 10.7717/peerj.19682 (PMC12296574; doi:10.7717/peerj.19682)

# WWTP

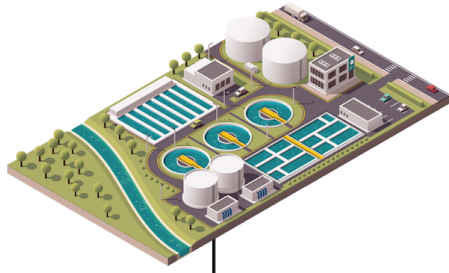

Influent

Sludge

Effluent

1. Bacterial Communities

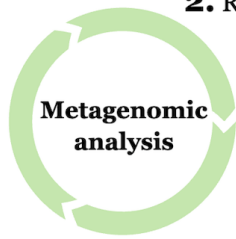

2. Resistome

3. Mobilome

1

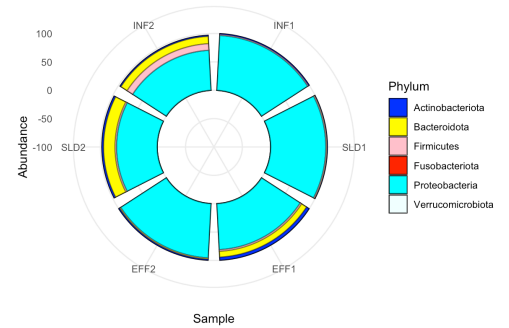

2

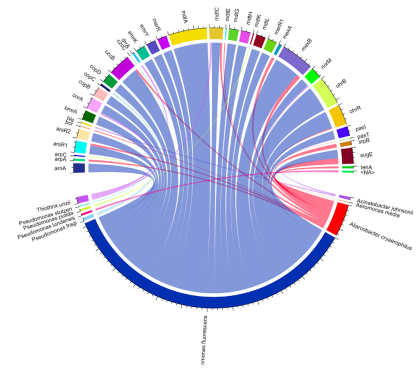

3

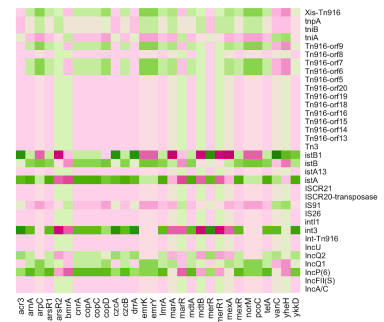

Supplement: Supplemental Information 2 [file peerj-13-19682-s002.pdf]
